# 7,8-DHF Modulates Aggressive Behavior in *Sebastes schlegelii*: Phenotype-Dependent Responses in Aggression-Dimorphic Individuals

**DOI:** 10.3390/ani15101463

**Published:** 2025-05-19

**Authors:** Shufei Xu, Xinna Ma, Yang Xiao, Tao Zhang, Chao Ma, Zhen Ma

**Affiliations:** 1 Key Laboratory of Environment Controlled Aquaculture, Ministry of Education, Dalian 116023, China; xsf0619@163.com (S.X.); maxinna@dlou.edu.cn (X.M.); xiaoyang@dlou.edu.cn (Y.X.); 2 College of Marine Technology and Environment, Dalian Ocean University, Dalian 116023, China; 3 Tianzheng Industrial Co., Ltd., Dalian 116021, China; zht_3000@163.com; 4 State Key Laboratory of Coastal and Offshore Engineering, Dalian University of Technology, Dalian 116081, China; machao@dlut.edu.cn; 5 College of Fisheries and Life Science, Dalian Ocean University, Dalian 116023, China

**Keywords:** 7,8-DHF, dose-dependent response, behavioral phenotyping, aggressive behavior, *Sebastes schlegelii*

## Abstract

Aggressive behavior is regulated by intricate neural circuits and molecular mechanisms, notably through the interaction of brain-derived neurotrophic factor (BDNF) with its receptor, tropomyosin receptor kinase B (TrkB), which influences neuroplasticity and related behavioral phenotypes. We investigate the role of the BDNF signaling pathway in fish aggression using juvenile black rockfish (*Sebastes schlegelii*), which exhibit distinct aggressive phenotypes. The TrkB agonist 7,8-dihydroxyflavone (7,8-DHF) was administered intraperitoneally at doses of 1.25, 2.5, and 5 mg/kg to assess its effects on the behavioral characteristics of high-aggression (H-agg) and low-aggression (L-agg) phenotypes. Our findings indicate the following: (1) The effects of 7,8-DHF are dose-dependent, with 2.5 mg/kg identified as the effective threshold dose for H-agg individuals; (2) in the H-agg group, this dose significantly reduced locomotor acceleration, angular velocity, and activity frequency, while prolonging the first movement latency; (3) in the L-agg group, only angular velocity was significantly decreased with the 2.5 mg/kg treatment, with no significant changes observed in other behavioral parameters. This study provides the first evidence for differential behavioral responses to 7,8-DHF in *S. schlegelii*, demonstrating dose-dependent aggression suppression in H-agg phenotypes and threshold-specific responses in L-agg phenotypes. These insights into the neuro-molecular basis of fish aggression can guide phenotype-specific management in aquaculture, potentially improving stress management, reducing injuries and mortality, and boosting productivity.

## Simple Summary

This study provides the first evidence that the TrkB receptor-specific agonist 7,8-dihydroxyflavone exerts differential regulatory effects on aggression phenotypes in *Sebastes schlegelii* through BDNF-TrkB signaling. Intraperitoneal administration of 2.5 mg/kg 7,8-DHF significantly reduced locomotor acceleration, angular velocity, and activity frequency in high-aggression individuals, while only decreasing angular velocity in low-aggression counterparts. These findings offer novel insights into the neural mechanisms underlying aggressive behavior and informs phenotype-based strategies for aquaculture optimization.

## 1. Introduction

Aggressive behavior constitutes an evolutionarily conserved strategy for resource competition, playing vital roles in habitat acquisition, mate selection, and reproductive success within natural ecosystems. However, under high-density intensive aquaculture conditions, the adverse effects of such behaviors are markedly amplified, thereby inducing compounded detrimental consequences [1]. Spatial confinement and restricted behavioral expression in captive environments often lead to frequent physical injuries, secondary infections, and impaired growth performance, collectively undermining both production efficiency and fish welfare standards. Notably, aggressive behavior exhibits considerable inter-individual variability within populations. Empirical research has shown that a minority (15–30%) of individuals with a H-agg phenotype account for over 80% of observed aggressive interactions [2]. This behavioral heterogeneity demonstrates significant environmental stability [3,4,5], as evidenced by studies on *Oncorhynchus kisutch* (coho salmon), which reveal consistent aggression profiles throughout extended acclimation periods [6]. The persistence of conserved aggression phenotypes strongly suggests that individual differences are primarily regulated by structural neuroplasticity (e.g., stable synaptic remodeling) rather than short-term environmental factors [7].

The maintenance of neuroplastic homeostasis critically depends on brain-derived neurotrophic factor (BDNF) [8], a key regulator of aggressive behavior through tyrosine kinase receptor B (TrkB)-mediated signaling [9,10,11]. Studies on *Mus musculus* (mice) and *Danio rerio* (zebrafish) have demonstrated that BDNF deficiency enhances neural circuit excitability and increases aggression, thereby revealing the evolutionarily conserved regulation of aggression by the BDNF-TrkB pathway [12,13]. The small-molecule TrkB agonist 7,8-dihydroxyflavone (7,8-DHF) effectively mimics BDNF’s neuroregulatory functions while circumventing the pharmacokinetic limitations associated with exogenous BDNF administration, such as its short half-life and poor blood–brain barrier permeability, which restrict its direct behavioral applications [14]. Mechanistically, 7,8-DHF modulates aggression through the activation of downstream MAPK/ERK signaling cascades and exhibits dose-dependent behavioral effects in mammalian models [15,16]. Nie et al. demonstrated the therapeutic potential of 7,8-DHF in ameliorating and even reversing behavioral deficits in mice [17]. Despite these advances, significant knowledge gaps persist regarding the behavioral effects of 7,8-DHF in piscine models. The dose–response relationships and phenotype-specific regulatory mechanisms under aquaculture conditions remain inadequately understood, particularly concerning individuals displaying distinct aggression phenotypes.

Accurate quantification of aggressive behavior is both a vital welfare indicator and a crucial metric for assessing neuromodulatory interventions [18,19]. In Atlantic salmon (*Salmo salar*), Oliveira et al. [20] demonstrated a positive correlation between chasing/nipping behaviors and fin damage. Individuals with high-aggression levels demonstrate significantly greater locomotor acceleration and reduced first movement latency, which have been established as quantitative indicators of aggression [21,22]. Importantly, these behavioral parameters correlate strongly with neural excitability pathways. The administration of fluoxetine, a selective serotonin reuptake inhibitor, significantly prolonged the latency to first movement in mice [23]. This behavioral modulation was closely associated with c-Fos expression, an immediate early gene marker of neuronal activation [24].

The black rockfish, *Sebastes schlegelii*, a species of significant ecological and economic importance in China, Korea, and Japan [25,26], exhibits frequent aggressive behavior that adversely affects production efficiency. Therefore, mitigating this behavior could improve profitability by reducing losses caused by injuries and stress. Recent research on the aggression of *S. schlegelii* has predominantly focused on environmental enrichment and physiological stress responses, while the neural mechanisms underlying its aggressive behavior remain unexplored [27,28,29]. This study aims to systematically investigate the behavioral responses of *S. schlegelii* with distinct aggression phenotypes to graded doses of 7,8-DHF, addressing three objectives: (1) determining the dose-dependent effects of 7,8-DHF on aggression modulation in juveniles; (2) identifying phenotype-specific differences in response thresholds to 7,8-DHF; (3) elucidating the regulatory patterns of 7,8-DHF across different aggression phenotypes. Our findings will enhance our understanding of the neurobiological basis of fish aggression and may reveal phenotype-dependent neural regulation mechanisms in aggressive behavior.

## 2. Materials and Methods

### 2.1. Experimental Fish and Husbandry

Juvenile *S. schlegelii* were obtained from a local hatchery (same genetic background) and maintained in the aquaculture system of the Aquaculture Facilities and Equipment Engineering Center. The recirculating aquaculture system (RAS) maintained all water quality parameters within optimal and stable ranges. Throughout the experimental period, water quality parameters were maintained as follows: water temperature was maintained at 20.0 ± 1.0 °C; salinity at 30 ± 1.0‰; an ammonia nitrogen concentration of 0.22–0.39 mg/L, and dissolved oxygen levels of 6.30–7.98 mg/L. A 12 h/12 h light/dark photoperiod was implemented. The fish were fed three times daily (08:30, 12:30, and 16:30) with commercial feed (2% of body weight per day; crude protein ≥ 44%, crude fat ≥ 5%). Prior to the experiments, the fish were acclimated for at least 7 days to stabilize their physiological conditions and fasted for 24 h. The experimental protocols were approved by the Dalian Ocean University Animal Ethics Committee (Approval No. 2023-47), and all procedures complied with China’s National Standards for Laboratory Animal Welfare (GB/T 35892-2018 and GB/T 35823-2018) [30,31].

### 2.2. Experimental Design

#### 2.2.1. Experimental Fish Tagging

The aggressive behavior experiment employed a random pairing method, ensuring a body weight difference of no more than 5% between paired fish to minimize size-related effects. A total of 48 healthy juveniles (11.12 ± 0.54 cm body length; 18.72 ± 0.92 g body weight) were selected. The fish were anesthetized 72 h prior to the trial using MS-222 (60 mg/L, Sigma-Aldrich, St. Louis, MO, USA), with the duration of anesthesia not exceeding 3 min. Colored plastic discs (d = 5 mm) were attached to the anterior dorsal fin using surgical sutures for identification purposes. The tagged fish were promptly reintroduced to their tanks, with strict adherence to the following criteria for the experimental subjects: the absence of hemorrhage, visible health effects, or abnormal behavior, and normal recovery of mobility within 60 s [32].

#### 2.2.2. Experimental Setup and Distinguishing Aggressive Phenotypes

**Figure 1:**
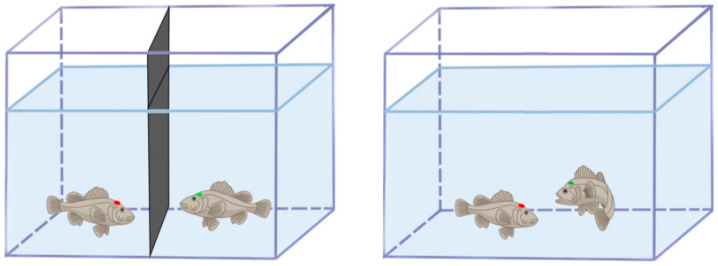
Experimental setup for dyadic confrontations. The high-aggression (H-agg) phenotype is denoted by red markings and the low-aggression (L-agg) phenotype is denoted by green markings.

The experimental tank (40 × 35 × 17.5 cm) contained a 10 cm water depth, with the sidewalls covered by white opaque stickers to minimize external disturbances. A high-definition camera (HIK-DS-2CD3T35D-I5, 1080P@30 fps, Hikvision, Hangzhou, China) was positioned vertically and recorded interactions for 10 min after partition removal (Figure 1) [21]. Aggression phenotyping was conducted using the real opponent paradigm, wherein two size-matched juveniles (ΔBW < 5%) were introduced into the experimental tank. This better mimicked natural ecological contexts, thereby eliciting ethologically relevant responses and enabling comprehensive quantification of aggressive phenotypes in both focal and opponent fish. Their interactions, including circling, chasing, biting, and withdrawal, were continuously monitored until the first attack–retreat sequence occurred, thereby identifying aggression-dimorphic individuals. Individuals dominating attacks and inducing opponent avoidance were classified as the H-agg phenotype, whereas those consistently exhibiting escape behaviors were designated as the L-agg phenotype. Aggressive behaviors were operationally defined as either sustained chasing (≥3 s/bout) by H-agg individuals toward L-agg individuals or body contact biting, which elicited escape responses in L-agg individuals.

#### 2.2.3. Experimental Fish Dosing

The experimental design included four groups: three treatment groups receiving 7,8-DHF at concentrations of 1.25, 2.5, and 5 mg/mL in a 30% DMSO-PBS vehicle, and a control group injected with an equivalent 30% DMSO-PBS. Each group consisted of six replicates (N = 6). The dosages were adjusted based on body weight (1.25, 2.5, 5 mg/kg) and administered intraperitoneally with a 100 μL injection using a self-refilling syringe (Socorex 174; Socorex, Ecublens, Switzerland). After a 72 h recovery period post-tagging, the injected fish were acclimated in the test tanks for 1 h before a 10 min behavioral recording session commenced.

### 2.3. Behavioral Analysis

All trials were video-recorded for 10 min to capture dyadic interactions and individual behavioral signatures. Automated trajectory tracking and parameter extraction were performed using EthoVision XT (v12.0, Noldus Information Technology, Wageningen, The Netherlands). Six behavioral metrics were analyzed: movement distance, locomotor acceleration, angular velocity, first movement latency, activity frequency, and immobility frequency.

Movement distance was quantified as the two-dimensional displacement (cm) of the fish centroid, calculated using the following formula:

$$D_{n}=\sqrt{{\left(X_{n}-X_{n-1}\right)}^{2}+{\left(Y_{n}-Y_{n-1}\right)}^{2}}$$

where $D_{n}$ represents the frame-to-frame displacement, and ($X_{n}$ − $X_{n-1}$) and ($Y_{n}\;$− $Y_{n-1}$) denote the coordinate differences along the X- and Y-axes between consecutive video frames (*n* and *n* − 1).

Locomotor acceleration was mathematically defined as the rate of change in the maximum velocity (cm/s^2^):

$$A_{n}\;=\frac{v_{n}-v_{n-1}}{t_{n}-t_{n-1}}$$

where $A_{n}$ indicates instantaneous acceleration, $v_{n}$ − $v_{n-1}$ represents the velocity difference between frames, and $t_{n}$ − $t_{n-1}$ is the time interval between frames.

Angular velocity was defined as the rate of change in the head direction (°/s):

$$W_{n}=\frac{T_{n}}{t_{n}-t_{n-1}}$$

where *w_n_* denotes the instantaneous turning rate, *T_n_* is the change in head orientation angle between frames, and $t_{n}$ − $t_{n-1}\;$is the sampling interval.

First movement latency was measured as the time (in seconds) from the removal of the partition until the initial displacement exceeded 1.0 cm.

Activity frequency was defined as a 20–60% change in body position between frames (captured at 10 frames per second), while immobility frequency was defined as a change of less than 20% in body position between frames (also at 10 frames per second).

### 2.4. Statistical Analysis

The data were expressed as arithmetic means ± SEM. All analyses were performed using Microsoft Office Excel 2019 and SPSS 21.0. The normality of the data was assessed using Shapiro–Wilk tests. One-way ANOVA was used to analyze the data that met the assumptions of normality and homoscedasticity, followed by Dunnett’s multiple comparison tests. In cases where heterogeneity of variance was detected, non-parametric tests (Kruskal–Wallis) were applied. To compare behavioral parameters between H-agg and L-agg individuals, we conducted two-tailed *t*-tests on normally distributed data (Shapiro–Wilk test, *p* > 0.05) with homoscedasticity (Levene’s test, *p* > 0.05)

## 3. Results

### 3.1. Aggressive Behavior Analysis in S. schlegelii

**Figure 2:**
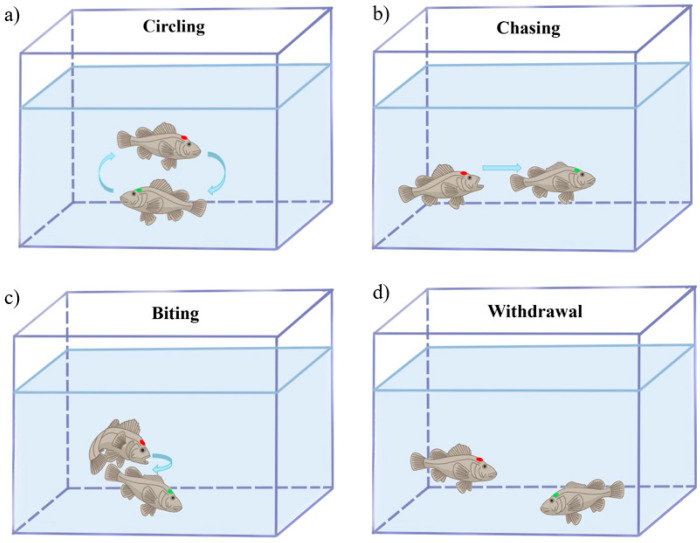
Graphical ethogram of aggressive behavior in *Sebastes schlegelii.* (**a**) Circular swimming; (**b**) aggressive chasing; (**c**) biting; (**d**) submission withdrawal. The high-aggression (H-agg) phenotype is denoted by red markings and the low-aggression (L-agg) phenotype is denoted by green markings.

In dyadic confrontations, H-agg and L-agg individuals exhibited distinct behavioral phenotypic divergence. Figure 2 illustrates the ethogram of aggressive behaviors in *S. schlegelii*. Circular swimming (Figure 2a) served as a mutual evaluation of combat potential. H-agg individuals initiated 93.60% of offensive acts, primarily manifesting as frequent chasing (Figure 2b) and targeted biting at caudal fins or lateral abdominal regions (Figure 2c). Conversely, L-agg individuals predominantly displayed escape behaviors, rapidly swimming away from attackers and occasionally displaying freezing responses, characterized by fin folding and immobility at the bottom of the tank (Figure 2d). Quantitative analysis of behaviors revealed that aggression constituted 16.84% of the total interaction time during the 10 min observation period. Specifically, chasing accounted for 88.29%, while biting comprised 11.71% of the aggressive interactions (Table 1).

### 3.2. Locomotor Parameters of Aggression-Dimorphic Individuals

A quantitative analysis of locomotor parameters across the high- and low-aggression phenotypes revealed significant group differences (Table 2). The H-agg individuals exhibited significantly greater locomotor acceleration compared to their L-agg counterparts (*p* < 0.01). The latency to first movement was substantially shorter in the H-agg individuals than in the L-agg individuals (*p* < 0.01). Conversely, the L-agg individuals exhibited a significantly higher immobility frequency than the H-agg individuals (*p* < 0.05). No significant differences were observed between the groups in terms of movement distance, angular velocity, or activity frequency (*p* > 0.05).

### 3.3. Effects of 7,8-DHF on Locomotor States in Aggression-Dimorphic Individuals

**Figure 3:**
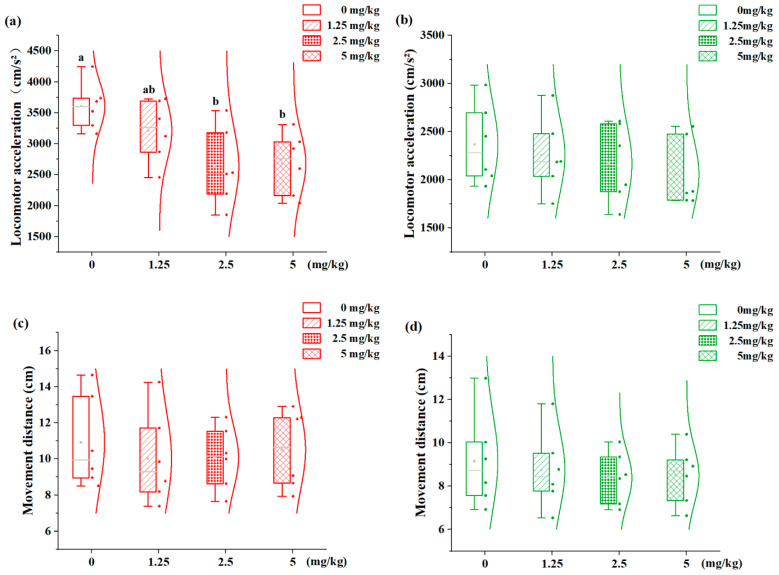
Effects of different concentrations of 7,8-DHF on the locomotor activity of H-agg and L-agg individuals. (**a**) Locomotor acceleration of H-agg individuals; (**b**) locomotor acceleration of L-agg; (**c**) movement distance of H-agg; (**d**) movement distance of L-agg. Values are presented as mean ± SE (n = 6). Different letters indicate significant differences (*p* < 0.05).

The concentration–response relationship of 7,8-DHF on the locomotor activity of fish with distinct phenotypes is shown in Figure 3. Movement distance and locomotor acceleration are considered fundamental aspects of locomotor behavior. The H-agg individuals displayed significant dose-dependent modulation in acceleration (Figure 3a). Specifically, both the 2.5 mg/kg (2633.18 cm/s^2^) and 5 mg/kg (2676.71 cm/s^2^) treatments resulted in significantly reduced acceleration compared to the controls (3607.54 cm/s^2^, *p* < 0.05), with no inter-dose differences (*p* > 0.05). In contrast, the L-agg juveniles exhibited similar locomotor acceleration across all 7,8-DHF concentrations compared to the 0 mg/kg controls (*p* > 0.05, Figure 3b). Movement distance was not significantly affected by the 7,8-DHF treatment in either phenotype (*p* > 0.05, Figure 3c,d).

### 3.4. Effects of 7,8-DHF on Spatial Distribution in Aggression-Dimorphic Individuals

**Figure 4:**
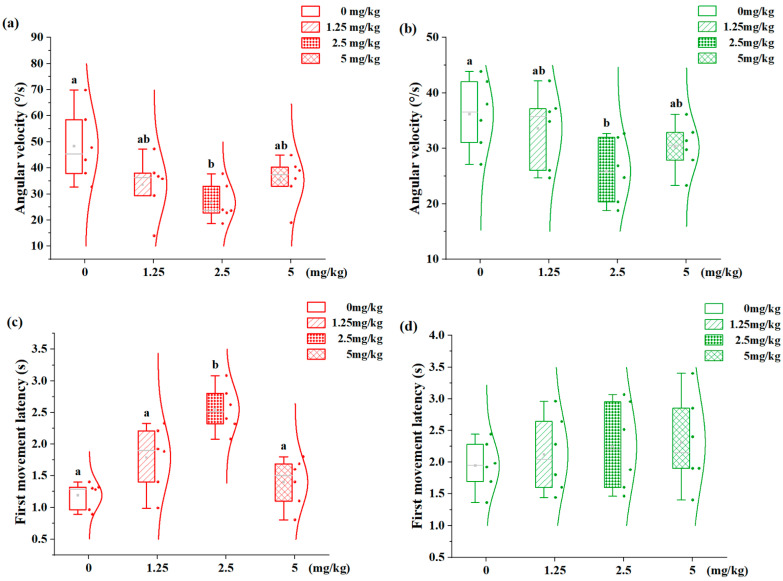
Effects of different concentrations of 7,8-DHF on the positional distribution of H-agg and L-agg individuals. (**a**) Angular velocity of H-agg; (**b**) angular velocity of L-agg; (**c**) first movement latency of H-agg; (**d**) first movement latency of L-agg. Values are presented as mean ± SE (n = 6). Different letters indicate significant differences (*p* < 0.05).

The concentration–response relationship of 7,8-DHF on spatial distribution patterns is illustrated in Figure 4. Angular velocity and first movement latency indicate spatial preference. The H-agg individuals demonstrated a dose-dependent reduction in angular velocity (Figure 4a). Specifically, the 2.5 mg/kg group (26.56°/s) showed significantly lower values than the control (0 mg/kg: 48.27°/s; *p* < 0.05), with no differences between the 1.25 and 2.5 mg/kg groups. Similarly, the L-agg individuals displayed reduced angular velocity at 2.5 mg/kg (25.85°/s vs. control 36.15°/s, *p* < 0.05), but no inter-group differences at other doses (Figure 4b). In contrast, the H-agg individuals showed dose-dependent increases in first movement latency (Figure 4c). The 2.5 mg/kg group (2.55 s) significantly surpassed all other groups (*p* < 0.05). No such effects were observed in the L-agg individuals across the tested concentrations (Figure 4d).

### 3.5. Effects of 7,8-DHF on Behavioral States in Aggression-Dimorphic Individuals

**Figure 5:**
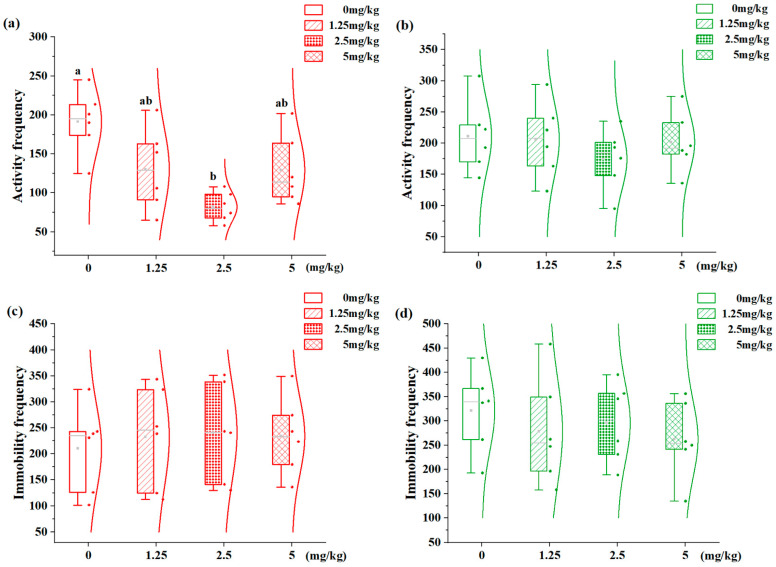
Effects of different concentrations of 7,8-DHF on the physical state of H-agg and L-agg individuals. (**a**) Activity frequency of H-agg; (**b**) activity frequency of L-agg; (**c**) immobility frequency of H-agg; (**d**) immobility frequency of L-agg. Values are presented as mean ± SE (n = 6). Different letters indicate significant differences (*p* < 0.05).

The dose–response relationship of 7,8-DHF on behavioral states in *S. schlegelii* is demonstrated in Figure 5. Activity frequency and immobility frequency serve as reliable metrics for assessing locomotor activity and behavioral modifications. The H-agg individuals exhibited a significant dose-dependent decrease in activity frequency (Figure 5a): the 2.5 mg/kg group (82.06 bouts) showed a 57.1% reduction compared to vehicle controls (0 mg/kg: 191.38 bouts; *p* < 0.05), with no effects at other concentrations (*p* > 0.05). The L-agg juveniles showed unchanged activity frequencies across all treatment groups (*p* > 0.05, Figure 5b). Immobility duration was not significantly altered in either phenotype at any tested concentrations (*p* > 0.05, Figure 5c,d).

## 4. Discussion

In dyadic confrontation, juvenile *S. schlegelii* individuals with high aggression and low aggression levels exhibited pronounced marked behavioral dimorphism, indicating a significant divergence in behavioral strategies. H-agg individuals initiated 93.6% of aggressive encounters, characterized by temporally clustered and highly repetitive attacks. These dominant fish predominantly occupied the central zone of the experimental arena, employing proactive approaching [21]. Conversely, L-agg individuals primarily exhibited demonstrated escape and freezing behaviors, showing a strong preference for the peripheral zone, consistent with passive defense mechanisms [33]. This center–periphery dichotomy corresponds to the bold–shy continuum reported in teleost personality studies [34]. Notably, aggressive interactions accounted for only 16.84% of the total observation time (Table 1), suggesting a pattern of “instantaneous high-intensity contest” in intraspecific competition. The chase-to-bite ratio (88.3% chasing vs. 11.7% biting) matched the energetically optimal ratio (4:1) reported in coral reef species [35]. Dominant H-agg individuals maintain social hierarchy through chase-intensive strategies, whereas subordinate L-agg individuals employ motion inhibition and submissive postures to avoid conflict. The greater prevalence of chasing over physical attacks, combined with ritualized circling and fin erection, reflects an evolutionary compromise between asserting dominance and conserving metabolic resources [36].

In the context of behavior classification, the mirror image test is a classical method that offers distinct advantages in terms of standardizing procedures and reducing the risk of physical injury to participants. Nevertheless, in order to elicit a more comprehensive expression of aggressive behaviors and complex behavioral sequences, this study employed the real opponent paradigm [37]. This methodological trade-off enabled the acquisition of more nuanced data through quantitative behavioral analysis. Quantitative analysis revealed that the H-agg individuals exhibited significantly higher locomotor acceleration and shorter first movement latency compared to their L-agg counterparts. These findings align with previous research [21,38], which demonstrates competitive advantages of rapid behavioral initiation in aggressive phenotypes. Notably, the lack of significant differences in movement distance and angular velocity indicates that acceleration and latency parameters more sensitively reflect motivational drive rather than overall locomotor capacity. The data support the neurobehavioral model that dissociates limbic-regulated attack motivation from motor circuit-dependent basic movement capacity [39].

This study provides the first evidence for differential behavioral modulation by 7,8-DHF across aggression phenotypes in *S. schlegelii*. The H-agg individuals exhibited significant dose-dependent reductions in acceleration (45%), angular velocity (28.5%), and activity frequency (57.1%) at optimal doses. TrkB, the high-affinity BDNF receptor, demonstrated increased activity in neural circuits associated with aggression [40], a pattern conserved in rodent and primate models [41,42]. Acting as a TrkB agonist, 7,8-DHF likely attenuates aggressive behaviors through enhanced BDNF/TrkB signaling in H-agg neural circuits. The bell-shaped dose–response curve suggests saturation of the TrkB binding site at 2.5 mg/kg, consistent with receptor occupancy pharmacodynamics [43]. The L-agg resistance to 7,8-DHF suggests fundamental differences in TrkB-expressing neuron ensembles between the phenotypes [44].

The impulsive aggressive responses of the H-agg phenotype appeared to be particularly sensitive to activity-dependent neuroplastic changes mediated by the TrkB signaling pathway. Pharmacological activation of TrkB induced rapid alterations in neuronal activity and neuromuscular transmission, impairing action potential conduction and contractile performance. This impairment is manifested as a reduction in acceleration among juvenile fish [45,46]. 7,8-DHF significantly decreased angular velocity and increased first movement latency in the H-agg phenotypes, without affecting movement distance, indicating a selective suppression of rapid response capability [43] rather than generalized motor function impairment. These effects align with the neuromodulatory action of 7,8-DHF on aggression-associated neural circuits, consistent with findings from mammalian models [47]. Parallel mechanisms occurred in dopamine signaling: D2 receptor antagonists reduced aggression but concurrently impaired motor function [48].

The differential effects of 7,8-DHF on H-agg and L-agg individuals suggest intrinsic differences in their neurotransmitter systems, potentially involving TrkB-dependent regulation of dopaminergic neuron plasticity. In H-agg individuals, excessive dopaminergic activity may drive aggressive behavior, as TrkB activation enhances synaptic plasticity in dopamine neurons [49]. The reduced locomotion in H-agg individuals reflects 7,8-DHF-induced downregulation of dopamine pathways, while L-agg individuals, characterized by lower basal dopamine activity, remained unaffected [50]. Furthermore, aggression phenotype differentiation is consistently associated with altered BDNF gene methylation, which likely underlie reduced basal TrkB signaling in L-agg individuals, thereby elevating their response threshold to exogenous agonists.

Notably, 7,8-DHF exhibited a biphasic (U-shaped) dose–response effect, with maximal suppression of movement initiation, turning speed, and locomotor activity observed in H-agg individuals at 2.5 mg/kg. This implies that supraoptimal TrkB stimulation activates compensatory inhibition mechanisms (e.g., via feedback loops or desensitization), attenuating aggression [51,52]. The suppressed activity and delayed movement initiation at this dose likely reflect metabolic trade-offs, where energy conserved from reduced aggression enhances stress adaptation. These behavioral changes are mediated by conserved neuroregulatory circuits, analogous to cortisol-driven modulation of energy metabolism. The consistency with canonical stress responses indicates that 7,8-DHF functionally emulates these adaptive pathways.

## 5. Conclusions

BDNF is pivotal in aggression regulation. Our findings show that 7,8-DHF (a TrkB agonist) effectively attenuates aggression in high-aggression *S. schlegelii* juveniles, with its U-shaped dose effect highlighting intricate neuroplasticity–receptor kinetic interplay in behavioral control. The critical dose of 2.5 mg/kg may represent a threshold where the drug’s action transitions from specific TrkB receptor activation to broader systemic interference. Subsequent research could explore BDNF-mediated aggression control through CNS expression analysis and mechanistic studies, offering solutions to mitigate fish aggression for enhanced welfare and aquaculture productivity. The present study employed one-way ANOVA to evaluate the independent effects of 7,8-DHF concentration on behavioral outcomes within each phenotype group, without examining potential concentration–phenotype interactions. Future investigations will specifically address these interaction effects to further elucidate 7,8-DHF’s behavioral modulation mechanisms.

## Figures and Tables

**Table 1:** Analyses of the duration of particular aggressive interactions (s).

	Ethogram of Aggressive Behavior	Time (s)	Proportion of Phenotypes %	Total Proportion %
Aggressive phenotype	Biting	11.83 ± 1.72	11.71	1.98
	Chasing	89.17 ± 6.23	88.29	14.86
Total		101	100	16.84
Non-aggressive phenotype	Circling	83.50 ± 3.43	16.73	13.91
	Withdrawal	415.50 ± 5.53	83.27	69.25
Total		499	100	83.16

**Table 2:** Behavioral parameters of H-agg and L-agg in the controls.

	Movement Distance (cm)	Locomotor Acceleration (cm/s^2^)	Angular Velocity (°/s)	First Movement Latency (s)	Activity Frequency	Immobility Frequency
H-agg	10.91 ± 1.04	3607.54 ± 156.35 **	48.27 ± 5.62	1.19 ± 0.09 **	191.38 ± 16.50	210.61 ± 33.77 *
L-agg	9.15 ± 0.89	2367.91 ± 169.29	36.15 ± 2.62	1.95 ± 0.16	210.91 ± 23.29	321.39 ± 33.91

Significant differences between H-agg and L-agg are denoted by asterisks (* indicates *p* < 0.05, ** indicates *p* < 0.01).

## Data Availability

The data supporting the findings of this study are available from the corresponding author upon justified request.
